# Self-reported skills and self-confidence in point-of-care ultrasound: a cross-sectional nationwide survey amongst Finnish emergency physicians

**DOI:** 10.1186/s12873-023-00795-w

**Published:** 2023-03-01

**Authors:** Ossi Hannula, Ville Hällberg, Anna Meuronen, Olli Suominen, Suvi Rautiainen, Ari Palomäki, Harri Hyppölä, Ritva Vanninen, Kalle Mattila

**Affiliations:** 1 School of Medicine, Institute of Clinical Medicine, University of Eastern Finland, Kuopio, Finland; 2 Emergency Department, Päijät-Häme Social and Health Care District, Lahti, Finland; 3 Emergency Department, Kanta-Häme Central Hospital, Hämeenlinna, Finland; 4 Emergency Department, Helsinki University Hospital, Porvoo, Finland; 5 Faculty of Medicine and Health Technology, Tampere University, Tampere, Finland; 6 Pihlajalinna Medical Centre Eastern Finland, Kuopio, Finland; 7 Emergency Department, South Savo Central Hospital, Mikkeli, Finland; 8 Department of Clinical Radiology, Kuopio University Hospital, Kuopio, Finland; 9 Emergency Department, Turku University Hospital, Turku, Finland; 10 Faculty of Medicine, University of Turku, Turku, Finland

**Keywords:** POCUS, Ultrasound, Post-graduate medical education, Continuous medical education, Skills training, Self-confidence

## Abstract

**Background:**

The use of point-of-care ultrasound (POCUS) is increasing. Numerous investigators have evaluated the learning curves in POCUS, but there are no published studies on how emergency physicians perceive their own competence level with this skill.

**Methods:**

A nationwide survey amongst Finnish emergency physicians was conducted. The respondents reported their use of POCUS and how it has affected their clinical decision-making. The number of POCUS examinations performed was compared to the self-assessed skill level with different applications. Cut-off values were determined for the number of examinations required to acquire a good self-assessed skill level in each POCUS application. The correlation between self-confidence and the self-estimated skill level was analyzed. Several different statistical methods were used, such as Student’s t-test, Pearson’s correlation test, Loess method and ROC curve analysis.

**Results:**

A total of 134 out of 253 Finnish emergency medicine specialists and residents (52%) responded to the survey. The most commonly used POCUS applications were POCUS-assisted procedures, pleural effusion and pneumothorax, inferior vena cava and lower extremity deep venous thrombosis. The initial rate of perceived skill acquisition was very steep with the curve flattening with greater skill and more experience. The number of examinations performed to reach a self-assessed good competence varied from seven to 75 with different applications. The lowest cut-off point for self-assessed good competence was obtained for rapid ultrasound for the shock and hypotension-protocol and the highest for focused cardiac examinations. There was an excellent correlation between self-confidence and the self-assessed skill level.

**Conclusions:**

The Finnish emergency practitioners’ self-assessed development of POCUS skills parallels the previously published learning curves of POCUS. The correlation of self-confidence and the self-assessed skill level was found to be excellent. These findings add information on the development of perceived POCUS skills amongst emergency physicians and could complement a formal performance assessment.

**Supplementary Information:**

The online version contains supplementary material available at 10.1186/s12873-023-00795-w.

## Introduction

Point of care ultrasound (POCUS) is often defined as the medical use of ultrasound (US) technology for the bedside evaluation of acute or critical medical conditions either in-hospital or in out-of-hospital settings. The term POCUS is used synonymously with other terms such as clinical, bedside, emergency, and physician-performed US. It is part of a larger field of clinical ultrasonography and is not meant to replace consultative US examinations [1].

The accuracy of multiple POCUS applications has been shown to be very good [2–5]. The value of POCUS is now gradually being recognized throughout the medical profession, as the use of POCUS has also been shown to improve patient satisfaction [6] and save health care resources [7, 8].

There are also concerns about the increasing use of POCUS, the most important being the quality of POCUS examinations [9]. POCUS, as with any other form of US, is heavily operator dependent and since the interpretation is made during the examination, it is necessary to be assured that the operator actually possesses the required skills.

An individual who is competent in POCUS should have the following capabilities 1) be able to identify when to perform a clinical US, 2) possess the technical skills required for US image and clip acquisition, 3) be capable of accurately interpreting US images, and 4) be able to incorporate the sonographic findings into clinical practice [10]. The number of performed POCUS examinations is often considered as a surrogate marker for competence. The American College for Emergency Physicians recommends 25–50 quality-reviewed POCUS exams for an individual application and a total of 150–300 POCUS exams in order to gain sufficient competence [1]. Although these numbers were originally based on expert opinions, there are now several studies supporting them [11–15]. Once a high level of skill has been acquired, it seems to be retained, although it might need continued training and supervision to maintain it at the optimal level [16].

A review of studies concerning the self-assessment of physicians has shown that physicians appear to have a limited ability to accurately self-assess, with the poorest accuracy evident among two groups - the least skilled and the most confident [17]. However, the self-assessed competence does seem to correlate with self-confidence [18–20] which has been shown to have a positive influence in adapting new skills [21]. The higher the self-confidence, the more likely that this will be reflected in higher POCUS use, which in turn enhances learning and potentially results in a better patient outcome [22]. As far as we are aware, there are no published studies evaluating how learners self-evaluate their POCUS skills.

The aim of this study was to acquire information on what POCUS examinations Finnish EM specialists and residents use in their clinical practice and how they self-assess their skills in each of them. Additionally, we aimed to estimate how many examinations were required to be completed in order to reach a good self-assessed competence in different POCUS applications. A nationwide survey was performed to achieve these aims.

## Materials and methods

### Study participants

Emergency medicine is a novel speciality in Finland. The first EM specialists trained according to the EM curriculum only graduated in 2016 (later termed as “specialists”). Based on the records maintained by the Finnish universities, at the end of January 2021, there were 56 specialists and 253 residents. These records include residents who are not working on their residency for various reasons. As the focus of the study was on the residents actively working in their field of emergency medicine, we retrospectively contacted all residents from the universities of Turku and Eastern Finland that had not yet graduated as specialists and asked whether they were working on their residency or planned to graduate as an EM specialist. The number of residents who were neither working in EM nor intending to graduate as an EM specialist was 20 (22%) out of 91 total residents in these universities. It was considered unlikely that these residents would answer this survey. Hence these individuals were excluded from the number of potential responders. These results were then extrapolated to the residents of all other universities resulting in a target group of 197 residents and therefore the total study population was 253 physicians.

### POCUS training

POCUS is considered an important skill for the EM physician. However, as the speciality is novel and rapidly developing, POCUS training has not been given the highest priority. Whereas in some hospitals the training has been somewhat structured, in other institutions the training has been virtually non-existent. Thus, learning about POCUS and putting it into practice has been largely dependent on an individual’s personal motivation on whether they participate in POCUS courses. The recently instituted formal definition of the skill set required for an EM specialist includes POCUS as a mandatory skill [23].

### Study design

The questionnaire was specifically designed for this study. It was based on previous publications [24, 25] and was supplemented with questions that were of special interest to the authors. A statistician experienced in designing surveys and three emergency medicine (EM) specialists with expertise in POCUS reviewed the survey to assess its clarity and relevance. We piloted the survey on a small group of EM specialists and modified the questionnaire based on their feedback. The final questionnaire (Additional file 1) comprised open and closed-ended questions related to demographics and primary occupation. The scope of POCUS applications was based on the European core curriculum for emergency medicine [26].

### Execution of the study

The survey was conducted in two phases. First, a pilot phase was conducted in autumn 2020 targeting EM residents and specialists in the four hospitals that had had the longest duration of EM training in Finland. Subsequently, the on-line survey targeted all of the other EM specialists and residents in Finland between Jan 15th and Feb 28th, 2021. The request to fill in the survey was distributed through the nationwide EM trainer working group, the membership list of the Finnish Society of Emergency Medicine, private social media groups, university records of EM residents and personal contacts of the research team.

Each respondent was provided with written information about the survey prior to answering the questions. Participating in the study was voluntary and answering the survey was considered as the provision of an informed consent. The survey does not have any direct identification data on the respondents and the Ethics committee of the Pirkanmaa hospital district approved the study.

### Methods

The respondents were asked to estimate the number of examinations that they had performed. They were also asked to self-assess their skill level in each of the 17 applications. Additionally, they reported how extensively they thought that the POCUS exams affected the diagnostic process. With respect to the skill level assessment and how much POCUS affects the diagnostic process, a five-point Likert scale was used (1 = very poor, 2 = poor, 3 = neither poor nor good, 4 = good, 5 = very good; there was also a sixth option 6 = do not perform the application in question).

We analyzed the correlation between the self-reported skill level and the physician’s self-rated confidence with his/her competence in that field. As a surrogate marker for the overall self-assessed skill level in POCUS, we calculated together the perceived competences of all the different applications. Thus, the numerical range of the overall skills extended from 0 to 85.

For the purposes of this study, the working places were categorized into three different categories: university hospital, secondary hospital, and other locations. This was thought to reflect the distinctions between the different hospitals such as the availability of radiologist services outside office hours. Thus, if the access to a radiologist was limited, this might increase the need for POCUS. Additionally, the distinction reflects the on-site availability of physicians from other specialities resulting in different skill requirements for EM physicians. The status of residency was subdivided into three categories: junior resident (residency less than 4 years), senior resident (residency 4 or more years), and specialists.

### Statistical analysis

The perceived self-assessed competence was compared to the self-reported number of examinations performed. The characteristics were expressed as means with standard deviations or frequencies and percentages. A locally weighted scatterplot smoothing method (loess) was used to generate a model for self-assessed skill acquisition for each individual type of examination. The scale of the number of examinations performed had a cut-off value at 200 and all points above that value are designated as 200.

The ROC curve analysis was used to find the cut-off point in the performed number of examinations in order to reach a self-assessed good competence for each POCUS application. For the purposes of this analysis, a dichotomous variable for self-assessed good competence was created. Self-assessed competence was defined as good when the self-assessed skill level was reported as Likert 4–5 (good or very good).

Student’s *t* test was used to compare means of overall self-assessed POCUS skill acquisition in different workplaces and at each stage of residency. Pearson’s correlation test was used to compare the self-assessed skill level of the different POCUS applications with respect to how much the POCUS examinations had affected the diagnostic process. It should be noted that not every respondent had performed all the various POCUS applications. All analyses are based on those respondents stating that they had actually performed that particular POCUS application.

## Results

The overall response rate was 53% (134/253). The response rates of specialists and residents were 68% (38/56) and 49% (96/197), respectively. The characteristics of the participants, presented in Table 1, show a slight male predominance (55%). The average age of specialists is only 39 years reflecting the novelty of the specialty. The most extensively used POCUS applications were POCUS assisted procedures, pleural effusion and pneumothorax (Thorax 1), inferior vena cava and lower extremity deep venous thrombosis, as shown in Table 2. Table 3 shows that the overall self-assessed skill level in POCUS improves as the physician progresses in her/his residency.

**Table 1:** Participants’ characteristics

Characteristic	Total (%)	Junior residents (%)	Senior residents (%)	Specialists (%)
Number of participants	134	56	35	38
Age (Years, SD)	36 (6.0)	32 (4.5)	37 (5.5)	39 (4.3)
Sex				
Male	73 (55%)	30 (54%)	17 (49%)	21 (58%)
Female	59 (44%)	26 (46%)	18 (51%)	15 (42%)
Occupation				
University hospital	49 (37%)	18 (33%)	15 (44%)	14 (37%)
Secondary hospital	69 (53%)	32 (58%)	15 (44%)	20 (53%)
Other	13 (10%)	5 (9%)	4 (12%)	4 (11%)
Uses ultrasound in his/her clinical work	126/132 (94%)	51/55 (93%)	34/35 (97%)	37/37 (100%)

Due to the varying numbers of non-responders and rounding up, all of the percentages might not equal 100%

**Table 2:** Terms and definitions of POCUS ultrasound applications

Term	Definition	Specialists *n* = 37	Residents *n* = 85	Total *n* = 126
Ultrasound assisted procedures	Includes all ultrasound assisted procedures	76% (*n* = 28)	79% (*n* = 67)	75% (*n* = 97)
Thorax 1	Pleural effusion and pneumothorax	76% (*n* = 28)	75% (*n* = 64)	73% (*n* = 92)
IVC	Assessment of inferior vena cava for volume load and central venous pressure	70% (*n* = 26)	71% (*n* = 60)	68% (*n* = 86)
Deep venous thrombosis	Limited compression ultrasound	68% (*n* = 25)	69% (*n* = 59)	67% (*n* = 84)
Focused cardiac examination	Pericardial fluid, left ventricular systolic function, enlargement of right ventricle	65% (*n* = 24)	68% (*n* = 58)	65% (*n* = 82)
FAST	Focus assessment with sonography for trauma	70% (*n* = 26)	65% (*n* = 55)	64% (*n* = 81)
Urinary system	Hydronephrosis, distended urinary bladder	68% (*n* = 25)	62% (*n* = 53)	62% (*n* = 78)
Gall bladder	Gallstones and cholecystitis	68% (*n* = 25)	58% (*n* = 49)	59% (*n* = 74)
Abdominal aorta	Abdominal aorta aneurysm	70% (*n* = 26)	56% (*n* = 48)	59% (*n* = 74)
Soft-tissue ultrasound	Foreign body, fluid collection/abscess, cellulitis	65% (*n* = 24)	49% (*n* = 42)	52% (*n* = 66)
Intrauterine pregnancy	Ruling-in pregnancy in uterus	62% (*n* = 23)	45% (*n* = 38)	48% (*n* = 61)
Thorax 2	Interstitial syndrome and pneumonia	51% (*n* = 19)	49% (*n* = 42)	48% (*n* = 61)
Musculoskeletal	Fracture, joint effusion, tendon injury	59% (*n* = 22)	33% (*n* = 28)	40% (*n* = 50)
RUSH protocol	Rapid ultrasound for shock and hypotension	51% (*n* = 19)	23% (*n* = 24)	34% (*n* = 43)
Ocular	Retinal detachment, elevated intracranial pressure	35% (*n* = 13)	19% (*n* = 16)	23% (*n* = 29)
Small bowel obstruction	Ruling-in small bowel obstruction	22% (*n* = 8)	16% (*n* = 14)	17% (*n* = 22)
BLUE protocol	Bedside lung ultrasound in emergency	5% (*n* = 2)	9% (*n* = 8)	8% (*n* = 10)

Modified from European core curriculum for EM [26]. The applications are presented from the most to the least extensively used. The number of specialists and residents performing the application in question are presented. The number represents the number of respondents that stated that they used the different applications of POCUS in their daily clinical work and is also presented as a percentage of all respondents in that group e.g. a total of 85 residents used some form of POCUS in their clinical work, 60 of them examined IVC with POCUS - the percentage is 60/85 = 71%. Due to the fact that not all respondents answered all subsections, the total number is higher than the sum of specialists and residents

**Table 3:** The overall self-assessed POCUS skills

Overall skill compared to the primary working place	
Hospital type	Mean overall skill (SD)
University hospital (*n* = 48)	36.0 (19.6)
Secondary hospital (*n* = 54)	33.7 (18.5)
Other (*n* = 9)	43.5 (20.1)
Total (*n* = 101)	35.4 (19.1)
Overall skill compared to the status of residency	
Status of residency	Mean overall skill (SD)
Junior residents (*n* = 48)	27.0 (18.2)
Senior residents (*n* = 24)	37.5 (13.8)
Specialists (*n* = 28)	49.0 (16.7)
Total (*n* = 100)	35.6 (19.1)

The acquired overall self-assessed POCUS skills consisting of the competences in each of the 17 individual applications (maximum 85), subdivided by the type of working place and the status of the residency. A total of 101 respondents gave details of their primary working place while 100 respondents described the status of their residency

The physicians were asked to answer only once to the questionnaire. We did not formally control that the orders were strictly followed. However, the background questions formed a profile and the answers formed a cluster for each individual participant. We did not recognize any identical profile and cluster combinations in our material.

**Fig. 1:**
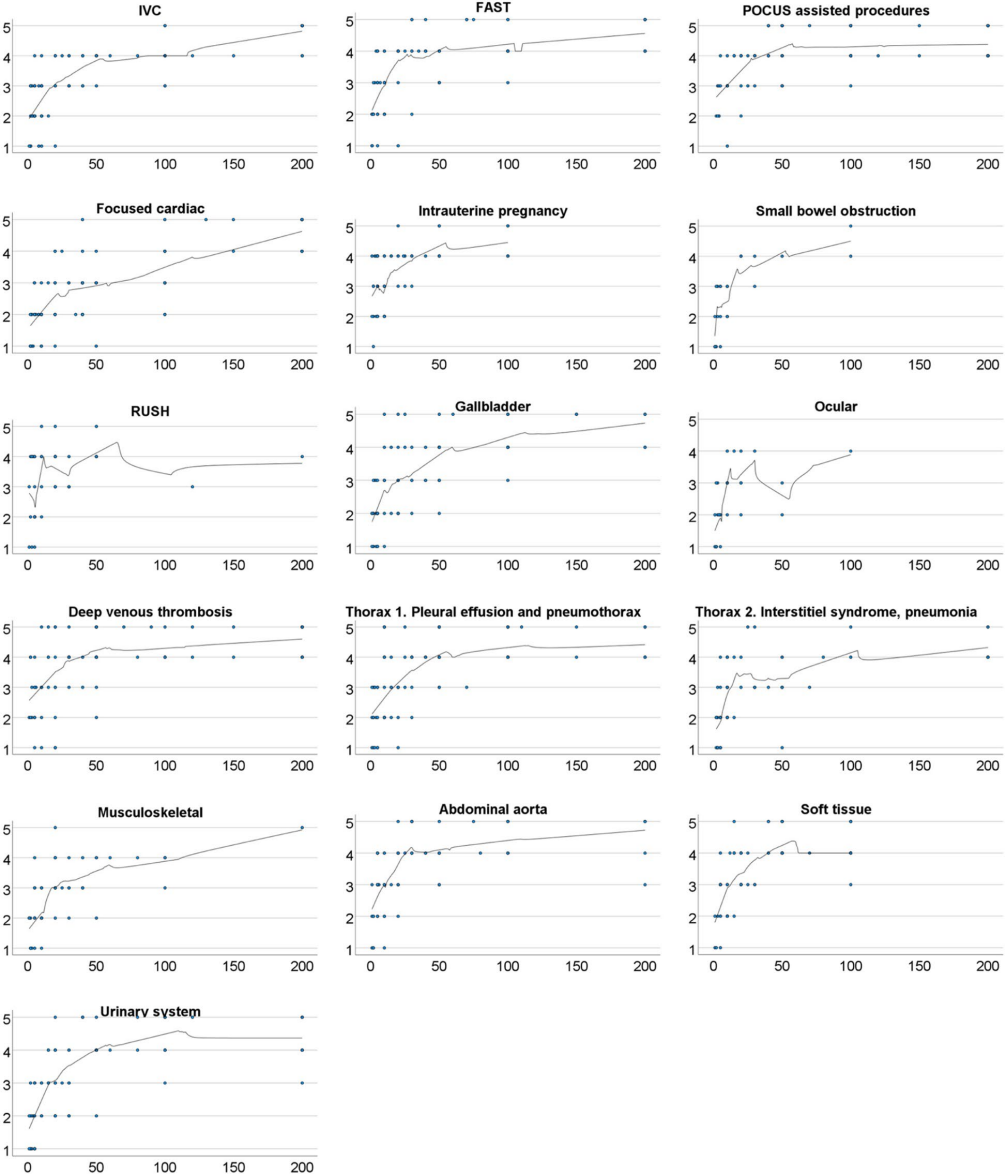
Self-perceived skill development in different applications of POCUS. The scale of the Y-axis in each curve is one to five on a Likert scale (1 = very poor, 2 = poor, 3 = neither poor nor good, 4 = good, 5 = very good) representing the self-assessed skill level and the scale of the X-axis is 0 to 200 reflecting the number of examinations performed. The scale was cut-off at 200 and all points above that are presented at that point

The self-rated assessments skill acquisition of the various POCUS applications are presented in Fig. 1 which shows that the perceived initial learning gradient is steep. When a higher self-assessed skill level is acquired, any additional learning requires much more experience. It is also evident that the actual gradient of the learning curve varies for the different applications. Results from applications with less than 20 respondents were considered unreliable and were excluded from further analysis.

The cut-off points for acquiring good self-assessed skills ranged from seven to 75 performed examinations. The focused cardiac exam was considered as the hardest in which to achieve self-assessed competency, while a focused assessment with sonography for trauma (FAST), intrauterine pregnancy, small bowel obstruction, ocular, thorax (pneumonia and interstitial syndrome) and musculoskeletal applications were considered as the easiest. A good perceived skill in rapid ultrasound for shock and hypotension (RUSH) protocol was found to be acquired with even fewer repetitions. The cut-off points for self-assessed good competence are presented in Table 4.

**Table 4:** Cut-off values for good perceived competence in different POCUS applications

POCUS application	Number of performed exams needed to acquire a good perceived competence: Cut-off values for good competence	Number of respondents
Ultrasound assisted procedures	27.5	95
Thorax 1 (pleural fluid, pneumothorax)	22.5	92
IVC	17.5	86
Deep venous thrombosis	25	84
Focused cardiac examination	75	82
FAST	15	81
Urinary system	35	78
Gall bladder	22.5	74
Abdominal aorta	17.5	74
Soft-tissue ultrasound	15	66
Intrauterine pregnancy	12.5	61
Thorax 2 (pneumonia, interstitial syndrome)	12.5	61
Musculoskeletal	15	50
RUSH protocol	7.5	43
Ocular	12.5	29
Small bowel obstruction	15	22

The number of repetitions needed to acquire a good perceived competence in different applications of POCUS. Cut-off points for perceived good competence were defined as self-reported skill of four to five (good or very good)

There was an excellent correlation between the self-assessed skill level and the physician’s self-confidence rating (*r* = 0.938, 95% CI 0.909 to 0.958, *p* < 0.001).

## Discussion

The self-assessed development of skills in POCUS among EM physicians shows a steep perceived learning curve at the beginning of training. This finding is consistent with previous data based on the expert assessment of recorded POCUS studies [11–13, 27]. The self-assessed rate of skills improvement became less steep with more experience and higher self-assessed skills. The cut-off points for perceived good competence ranged between seven to 75 exams performed. The cutoff values are related to self-assessed skills, not to absolute competence. While the previous evidence on cut-off values for competency in POCUS is limited, this range of values parallels with most of the published literature [11, 13, 14, 27, 28]. These findings indicate that Finnish EM physicians’ estimation of their POCUS skills acquisition seems to be plausible.

Gaspari et al. assessed how clinical sonographers interpreted gallbladder US images on a digital videotape with the tapes being later reviewed by two expert sonographers with respect to interpretation, image acquisition and image quality [11]. In that study, 25 repetitions were found to be sufficient to acquire a clinical competency while in the current study the value was found to be 22. Jang et al. described a cut-off value of 30 for diagnosing hydronephrosis reliably with POCUS, while in our study the value was 35 [14]. In a retrospective review of a US database, various POCUS examinations performed by EM residents and physicians were recorded. The recordings were then evaluated by physicians who were experts in POCUS, in terms of image interpretation, image acquisition and image quality. The cut-off values for good competence in this study were higher than in other published literature as the authors estimated that between 27 up to 90 repetitions would be needed to reach a plateau for interpreting an image correctly and an average of 50 exams were found to reach a reasonable performance level comparable to that of expert sonographers [12].

The ROC curve analysis shows the best approximation on the number of exams required to achieve good self-reported competence; after that value, the rate of learning plateaus. In some of the applications the loess curve was again lowered after the cut-off point. In these applications, there were a limited number of respondents reporting a higher number of performed exams than the cut-off value. Hence the curve would be highly affected by even a single response. Slightly lower accuracy of POCUS in individuals gaining more experience has been published before [12]. One possible explanation is that with more experience and more skill, the physician may attempt to use the POCUS application in more complex settings resulting in a higher rate of failures. It is highly unlikely that the skills would deteriorate with increasing experience.

A focused cardiac examination displayed the highest cut-off point for perceived good skill in our study. No previous investigations were found where the performance of a focused cardiac examination by emergency physicians has been assessed. In the literature, a cardiac examination has been suggested to be one of the easiest applications to reach a competence plateau when the sole focus was on identifying the presence of pericardial effusion [12]. Nonetheless, when the focus was more complex, such as in this study, both acquiring the correct acoustic windows and interpreting the results often became more difficult. We determined the cut-off point for self-reported good competence to be 75 examinations. As the focused cardiac exam consists of only the basic applications of cardiac ultrasound, this seems reasonable when compared to a recommendation issued by the American College of Cardiology that the physician should perform a minimum number of 150 examinations, before he/she can be considered competent to conduct a comprehensive echocardiological assessment [29].

The least used POCUS applications in this study were small bowel obstruction, ocular, RUSH, musculoskeletal, intrauterine pregnancy, interstitial syndrome and pneumonia (Thorax 2), soft-tissue ultrasound and bedside lung ultrasound in emergency (BLUE) protocol. These applications have not usually been taught to physicians new to POCUS. It is likely that the residents have initially learned some basic applications, such as deep venous thrombosis, FAST and gallbladder, and then later expanded their knowledge to these other applications and this might explain why fewer physicians were performing these applications. As those physicians willing to adopt these applications probably already possess the basic POCUS skills, they are arguably able to learn new applications more easily, resulting in lower cut-off values for good self-reported competence.

The ROC curve analysis for interstitial syndrome and pneumonia (Thorax 2) revealed a cut-off value of 12.5 after which the curve stabilized - a value somewhat lower than expected. A possible explanation would be that the question had two applications, interstitial syndrome and pneumonia, of which the first one is probably more often used and better understood, and some respondents might have considered their skill level based on only that diagnosis.

One of the studied POCUS applications was RUSH, which is a POCUS protocol for patients with hypotension. It consists of several POCUS evaluations: abdominal aorta aneurysm, inferior vena cava, focused assessment with sonography for trauma, deep venous thrombosis, focused cardiac examination, pneumothorax and intrauterine pregnancy. In this study, the cut-off value to achieve a self-assessed good skill was 7.5 with the loess curve displaying a steep learning gradient with low experience. It is likely that many of the respondents first learn certain individual POCUS studies. When these skills are already adopted, combining them as a standard protocol seems to be rather quickly embraced.

The correlation between self-assessed skill level and self-confidence was excellent, indicating that the individual’s self-confidence increased rapidly with training. This finding is consistent with previous studies focusing on other skills [18, 20, 30]. It seems that the cut-off point for good self-confidence parallels the cut-off point for good self-assessed competence.

Junior residents were the largest respondent group in this study, a fact that reflects the population of EM residents and specialists in Finland. As EM is a young speciality, many graduate physicians have entered into a residency but rather few have completed it already. The proportion of junior residents was highest in secondary hospitals. Residency is most often started in a secondary hospital and finished in a university hospital which explains this finding.

The overall self-assessed POCUS skills increased with the progression of the residency. Junior residents had a self-assessed skill level of 27.0, for senior residents it was 37.5 and for specialists it was even higher, 49.0 i.e. this progression seems logical. There was also a minor difference in overall self-assessed skills related to the workplaces. The overall self-assessed skills were lowest in the secondary hospitals (33.7), consistent with the highest proportion of junior residents in these institutions as compared to university hospitals (36.0) and other locations (43.5) but it should be noted that these differences are rather minor.

This nationwide survey provides valuable information on the use and development of self-assessed skills and self-confidence in POCUS among Finnish EM physicians, which can be viewed as the development of a novel skill within a new medical speciality. The residency has lately been shifting from being time-based to a competence-based assessment both in Finland and in multiple other European countries. In Emergency Medicine, POCUS is named as one of the 24 key skills to be evaluated throughout the residency. Nevertheless, also the competence-based residency includes a self-assessment of competency in addition to the evaluation by a supervisor. The results of this study indicate that the accumulation of self-confidence and self-assessed skill level exhibit a high correlation, and the cut-off points for the number of examinations required to achieve good self-assessed competence seem to parallel the cut-off points of good competence as assessed by an expert supervisor in previous studies [11, 13, 14, 27]. This finding adds information on perceived POCUS skill development amongst emergency physicians and could complement a formal performance assessment. In a resource-limited setting in which a formal accreditation is not available, these findings may help in determining when a physician is competent to perform POCUS in his/her clinical work.

### Limitations

This study does not come without limitations. The data reported is based on retrospective self-assessments. It is impossible for an individual physician to remember the exact number of exams performed throughout his/her whole career. It has been previously shown that also physicians have a limited capacity for self-assessment [17]. However, the goal of this study was to compare the self-assessment to previously published learning curves for various POCUS applications in order to gain information to complement other methods of skills’ evaluation.

The response rate of 53% was moderate. The university records of residents include multiple individuals who are no longer working in EM and do not intend to finish their residency but have not yet switched to another speciality. Additionally, the university records include residents that are currently not working in EM e.g. due to maternity leave or military service i.e. individuals that are very hard to contact. In order not to overestimate the response rate, we included all the residents that we were not able to reliably exclude as potential respondents. As the questionnaire was directed to the residents actively working on their residency, the true response rate is most likely somewhat higher than that reported. The specialists, on the other hand, responded rather well with a response rate of 68%. As a whole, the response rate was somewhat higher than the response rates of other recent surveys targeting physicians [31, 32]. It is possible that this response rate may lead to a non-response bias. Nonetheless, the demographics of the participants revealed a wide range of occupation, age, and status of residency and that should limit the possibility of non-respondent bias. In addition, a recent study suggested that the non-respondent bias might not be as influential as previously thought [33].

The number of respondents restricted our possibility to conduct subgroup analyses. In fact, trying to draw reliable conclusions from the self-assessed learning curves on applications with less than 50 respondents might have limited value. Furthermore, in most applications the number of respondents who reported over 100 exams, was low.

However, while one may question the validity of the complete self-assessed learning curves in the different POCUS applications, overall, the results of this study illustrate rather well the perceived initial learning of POCUS skills amongst emergency physicians.

## Conclusions

The self-assessed development of skills in POCUS amongst emergency physicians and residents parallels the previously published POCUS learning curves based on expert reviews of image acquisition and interpretation. Not surprisingly, there are variations in the cut-off values for perceived good competence in different POCUS applications.

## Supplementary Information

**Additional file 1.**

## Data Availability

The data that support the findings of this study are available from the corresponding author upon reasonable request.

## Abbreviations

POCUS: Point-of-care ultrasound
US: Ultrasound
EM: Emergency medicine
IVC: Inferior vena cava
BLUE: Basic lung ultrasound in emergency
FAST: Focused assessment with sonography for trauma
RUSH: Rapid ultrasound for shock and hypotension

## References

[1] ACEP (2016). Ultrasound guidelines: emergency, point-of-care, and clinical ultrasound guidelines in medicine.

[2] Staub LJ, Mazzali Biscaro RR, Kaszubowski E, Maurici R (2019). Lung ultrasound for the emergency diagnosis of pneumonia, acute heart failure, and exacerbations of chronic obstructive pulmonary disease/asthma in adults: a systematic review and meta-analysis. J Emerg Med.

[3] Pourmand A, Dimbil U, Drake A, Shokoohi H (2018). The accuracy of point-of-care ultrasound in detecting small bowel obstruction in emergency department. Emerg Med Int.

[4] Keikha M, Salehi-Marzijarani M, Soldoozi Nejat R, Sheikh Motahar Vahedi H, Mirrezaie SM (2018). Diagnostic accuracy of rapid ultrasound in shock (RUSH) exam; a systematic review and meta-analysis. Bull Emerg Trauma.

[5] Ross M, Brown M, McLaughlin K, Atkinson P, Thompson J, Powelson S (2011). Emergency physician-performed ultrasound to diagnose cholelithiasis: a systematic review. Acad Emerg Med.

[6] Howard ZD, Noble VE, Marill KA, Sajed D, Rodrigues M, Bertuzzi B (2014). Bedside ultrasound maximizes patient satisfaction. J Emerg Med.

[7] Lentz B, Fong T, Rhyne R, Risko N (2021). A systematic review of the cost-effectiveness of ultrasound in emergency care settings. Ultrasound J.

[8] Hannula O, Vanninen R, Rautiainen S, Mattila K, Hyppölä H (2021). Teaching limited compression ultrasound to general practitioners reduces referrals of suspected DVT to a hospital: a retrospective cross-sectional study. Ultrasound J.

[9] Chawla TP, Cresswell M, Dhillon S, Greer M-LC, Hartery A, Keough V (2019). Canadian Association of Radiologists position statement on point-of-care ultrasound. Can Assoc Radiol J.

[10] Damewood SC, Leo M, Bailitz J, Gottlieb M, Liu R, Hoffmann B (2020). Tools for measuring clinical ultrasound competency: recommendations from the ultrasound competency work group. AEM Educ Train.

[11] Gaspari RJ, Dickman E, Blehar D (2009). Learning curve of bedside ultrasound of the gallbladder. J Emerg Med.

[12] Blehar DJ, Barton B, Gaspari RJ (2015). Learning curves in emergency ultrasound education. Burton JH, editor. Acad Emerg Med.

[13] Kim J, Kim K, Kim J, Yoo J, Jeong W, Cho S (2018). The learning curve in diagnosing acute appendicitis with emergency sonography among novice emergency medicine residents. J Clin Ultrasound.

[14] Jang TB, Jack Casey R, Dyne P, Kaji A (2010). The learning curve of resident physicians using emergency ultrasonography for obstructive uropathy. Acad Emerg Med.

[15] Carrié C, Biais M, Lafitte S, Grenier N, Revel P, Janvier G (2015). Goal-directed ultrasound in emergency medicine: evaluation of a specific training program using an ultrasonic stethoscope. Eur J Emerg Med.

[16] Schleifer J, Haney RM, Shokoohi H, Huang CK, Ratanski D, Kimberly H (2021). Longitudinal accuracy analysis of ultrasound performed during a four-year emergency medicine residency. AEM Educ Train.

[17] Davis DA, Mazmanian PE, Fordis M, Van Harrison R, Thorpe KE, Perrier L (2006). Accuracy of physician self-assessment compared with observed measures of competence: a systematic review. JAMA.

[18] Bauman B, Kernahan P, Weinhaus A, Walker MJ, Irwin E, Sundin A (2021). An interprofessional senior medical student preparation course: improvement in knowledge and self-confidence before entering surgical training. Adv Med Educ Pract.

[19] Johnson J, Stromberg D, Williams B, Greenberg N, Myers O (2021). Point-of-care ultrasound for family medicine residents: attitudes and confidence. Fam Med.

[20] Stolz LA, Amini R, Situ-LaCasse E, Acuña J, Irving SC, Friedman L, et al. Multimodular ultrasound orientation: residents’ confidence and skill in performing point-of-care ultrasound. Cureus. 2018;10(11) Available from: https://www.cureus.com/articles/15041-multimodular-ultrasound-orientation-residents-confidence-and-skill-in-performing-point-of-care-ultrasound. Cited 2022 Apr 5. 10.7759/cureus.3597 PMC6338403 30680258

[21] Vergara-Rodríguez D, Antón-Sancho Á, Fernández-Arias P (2022). Variables influencing professors’ adaptation to digital learning environments during the COVID-19 pandemic. Int J Environ Res Public Health.

[22] Mutlu GY, Eren E, Eviz E, Gokce T, Sakarya S, Hatun S. The attitudes, experiences, and self-competencies of pediatric endocrinology fellows and attending physicians regarding diabetes technology: the Turkey experience. J Pediatr Endocrinol Metab. 2022. 10.1515/jpem-2022-0024. 10.1515/jpem-2022-0024 35334193

[23] Finnish Universities. Osaamisen arviointi Erikoislääkärikoulutus (in Finnish). Available from: https://www.laaketieteelliset.fi/site/files/ammatillinen-jatkokoulutus-dokumentit/Opinto-oppaat/Valtakunnalliset%20opinto-oppaat/EL_Osaamisen%20arviointi_2021-2022.pdf. Cited 2022 Apr 8.

[24] Kim DJ, Theoret J, Liao MM, Kendall JL (2014). Experience with emergency ultrasound training by Canadian emergency medicine residents. West J Emerg Med.

[25] Leschyna M, Hatam E, Britton S, Myslik F, Thompson D, Sedran R, et al. Current state of point-of-care ultrasound usage in Canadian emergency departments. Cureus. 2019;11(3) Available from: https://www.ncbi.nlm.nih.gov/pmc/articles/PMC6516619/. Cited 2021 Feb 12. 10.7759/cureus.4246 PMC6516619 31131169

[26] EuSEM (2019). European core curriculum for Emergency Medicine. Version 2.0.

[27] Hansen W, Mitchell CE, Bhattarai B, Ayutyanont N, Stowell JR (2017). Perception of point-of-care ultrasound performed by emergency medicine physicians. J Clin Ultrasound.

[28] Rankin JH, Elkhunovich MA, Rangarajan V, Chilstrom M, Mailhot T. Ultrasound in Emergency Medicine Learning curves for ultrasound assessment of lumbar puncture insertion sites: when is competency established?. 10.1016/j.jemermed.2016.03.025 Cited 2022 Sep 26. 10.1016/j.jemermed.2016.03.025 27231207

[29] Wiegers SE, Ryan T, Arrighi JA, Brown SM, Canaday B, Damp JB (2019). 2019 ACC/AHA/ASE advanced training statement on echocardiography (revision of the 2003 ACC/AHA clinical competence statement on echocardiography): a report of the ACC Competency Management Committee. Catheter Cardiovasc Interv.

[30] Mercuzot C, Debien B, Riviere É, Martis N, Sanges S, Galland J (2021). Impact of a simulation-based training on the experience of the beginning of residency. Rev Méd Interne.

[31] Wiebe ER, Kaczorowski J, MacKay J (2012). Why are response rates in clinician surveys declining?. Can Fam Physician.

[32] Cunningham CT, Quan H, Hemmelgarn B, Noseworthy T, Beck CA, Dixon E (2015). Exploring physician specialist response rates to web-based surveys. BMC Med Res Methodol.

[33] Hendra R, Hill A (2019). Rethinking response rates: new evidence of little relationship between survey response rates and nonresponse bias. Eval Rev.

