# Supplementary material for: Self-reported skills and self-confidence in point-of-care ultrasound: a cross-sectional nationwide survey amongst Finnish emergency physicians
Source: BMC Emerg Med. 2023 Mar 1;23:23. doi: 10.1186/s12873-023-00795-w (PMC9979460; doi:10.1186/s12873-023-00795-w)
Supplement: Supplementary file 1 — Additional file 1. [file 12873_2023_795_MOESM1_ESM.pdf]

EMERGENCY PHYSICIAN PERFORMED POINT-OF-CARE ULTRASOUND

Tampere University

Survey for emergency medicine residents and specialists

Background information

Age (years)
________________ v.

Sex (male/female/other/I do not wish to tell)
1. Male
2. Female
3. Other / I do not wish to tell

MD graduation year
____

Years in emergency medicine residency program
____

Emergency medicine specialist
1. Yes
2. No

Primary occupation
Name of the hospital ____

Number of hours of clinical work per week
_______________h

Specialist in other specialities?
1, Yes
2. No
3. Which ones? ______________________________________

Do you use ultrasound in your work?
1. Yes
2. No

If yes – continue to question 1.

If no, answer the following question. Please choose one or more choice and provide an open answer.

Why not?
1 .I don't know how
2 .I don't have time
3. I prioritize other methods
4. I don't trust ultrasound performed by non-radiologist
5. I only treat patients that do not benefit from ultrasound
6. Other reasons, please specify____

____

Which factors would make you to start using ultrasound? ____

____

____

1. Own attitude on starting to perform a new ultrasound application? Please put a sign in the correct place on the line.

I want to understand an application thoroughly before incorporating it in clinical setting.	|———————————|	Basics should be learned beforehand, but the actual skill is learned in clinical setting.

2. How much ultrasound training have you received during your residency? Choose one.

1 Not at all
2 A little
3 Neither little nor much
4 Much
5 Very much

3. My ultrasound training has been (Place state a number and circle the most appropriate answer on every line.)

Type of ultrasound training	Training or self-learning during residency	This type of training has helped me to use ultrasound in my clinical setting.				
	Hours per month on average.	Strongly disagree	Disagree	Neither agree nor disagree	Agree	Strongly agree
Reading books or scientific articles	(h/month)	1	2	3	4	5
Following webpages, blogs, podcasts	(h/month)	1	2	3	4	5
Bedside teaching by a senior physician	(h/month)	1	2	3	4	5
Bedside practicing, supervised by a senior physician	(h/month)	1	2	3	4	5
Independent practice	(h/month)	1	2	3	4	5

Type of ultrasound training	Training or self-learning during residency	This type of training has helped me to use ultrasound in my clinical setting.				
	Days per year on average.	Strongly disagree	Disagree	Neither agree nor disagree	Agree	Strongly agree
Didactic lectures.	(h/month)	1	2	3	4	5
Structured training on your own workplace.	(h/month)	1	2	3	4	5
Training on phantoms.	(h/month)	1	2	3	4	5
Dedicated ultrasound courses outside you own workplace.	(h/month)	1	2	3	4	5

4. Please answer the following questions

Ultrasound application	Which of the following applications you have received training for? Answer every line.		How many times have you performed the application in question? Estimate a number.	Do you perform the application routinely? Answer every line.	
	Yes	No		Yes	No
Inferior vena cava	1	0		1	0
BLUE protocol	1	0		1	0
FAST protocol	1	0		1	0
Ultrasound assisted procedures	1	0		1	0
Focused cardiac ultrasound	1	0		1	0
Intrauterine pregnancy	1	0		1	0
Small bowel obstruction	1	0		1	0
RUSH protocol	1	0		1	0
Cholelithiasis and cholecystitis	1	0		1	0
Ocular	1	0		1	0
Deep venous thrombosis (limited)	1	0		1	0
Thorax I (pleural effusion and pneumothorax)	1	0		1	0
Thorax 2 (decompensated heart failure and pneumonia)	1	0		1	0
Musculoskeletal (joint effusion, fractures, tendon injuries)	1	0		1	0
Abdominal aorta aneurysm	1	0		1	0
Foreign body, cellulitis, abscess	1	0		1	0
Urinary system (hydronephrosis and urinary retention)	1	0		1	0

5. Please assess your skill in each of the following ultrasound applications

Ultrasound application	How good I think is my skill in performing the following applications? Answer every line.					
	Very poor	Poor	Neither poor nor good	Good	Very good	Do not perform the application in question
Inferior vena cava	1	2	3	4	5	0
BLUE protocol	1	2	3	4	5	0
FAST protocol	1	2	3	4	5	0
Ultrasound assisted procedures	1	2	3	4	5	0
Focused cardiac ultrasound	1	2	3	4	5	0
Intrauterine pregnancy	1	2	3	4	5	0
Small bowel obstruction	1	2	3	4	5	0
RUSH protocol	1	2	3	4	5	0
Cholelithiasis and cholecystitis	1	2	3	4	5	0
Ocular	1	2	3	4	5	0
Deep venous thrombosis (limited)	1	2	3	4	5	0
Thorax I (pleural effusion and pneumothorax)	1	2	3	4	5	0
Thorax 2 (decompensated heart failure and pneumonia)	1	2	3	4	5	0
Musculoskeletal (joint effusion, fractures, tendon injuries)	1	2	3	4	5	0
Abdominal aorta aneurysm	1	2	3	4	5	0
Foreign body, cellulitis, abscess	1	2	3	4	5	0
Urinary system (hydronephrosis and urinary retention)	1	2	3	4	5	0

6. The ultrasound applications affecting my clinical decision making. Please evaluate the current effect.

Ultrasound application	How big effect the ultrasound exam I perform has on my clinical decision making? Answer every line.					
	Very poor	Poor	Neither poor nor goof	Good	Very good	Do not perform the application
Inferior vena cava	1	2	3	4	5	0
BLUE protocol	1	2	3	4	5	0
FAST protocol	1	2	3	4	5	0
Ultrasound assisted procedures	1	2	3	4	5	0
Focused cardiac ultrasound	1	2	3	4	5	0
Intrauterine pregnancy	1	2	3	4	5	0
Small bowel obstruction	1	2	3	4	5	0
RUSH protocol	1	2	3	4	5	0
Cholelithiasis and cholecystitis	1	2	3	4	5	0
Ocular	1	2	3	4	5	0
Deep venous thrombosis (limited)	1	2	3	4	5	0
Thorax I (pleural effusion and pneumothorax)	1	2	3	4	5	0
Thorax 2 (decompensated heart failure and pneumonia)	1	2	3	4	5	0
Musculoskeletal (joint effusion, fractures, tendon injuries)	1	2	3	4	5	0
Abdominal aorta aneurysm	1	2	3	4	5	0
Foreign body, cellulitis, abscess	1	2	3	4	5	0
Urinary system (hydronephrosis and urinary retention)	1	2	3	4	5	0

7. In which of the following ultrasound guided procedures have I received training for?

Procedure	Theoretical training		Hands-on training (on phantoms, tissue samples, patients)	
	Yes	No	Yes	No
Arterial cannulation	1	0	1	0
Paracentesis	1	0	1	0
Central vein cannulation	1	0	1	0
Lumbar punction	1	0	1	0
Abscess draining	1	0	1	0
Joint draining	1	0	1	0
Peripheral vein cannulation	1	0	1	0
Pericardiocentesis	1	0	1	0
Peritonsillar abscess draining	1	0	1	0
Thoracentesis	1	0	1	0
Nerve blocks	1	0	1	0
Suprapubic catheterization	1	0	1	0
Deep abscess draining	1	0	1	0
Pacemaker installation	1	0	1	0
Foreign body removal	1	0	1	0

8. How many procedures have you performed? Estimate. Do you perform them routinely?

Procedure	Estimation on the number of procedures performed	I perform this procedure routinely	
		Yes	No
Arterial cannulation		1	0
Paracentesis		1	0
Central vein cannulation		1	0
Lumbar punction		1	0
Abscess draining		1	0
Joint draining		1	0
Peripheral vein cannulation		1	0
Pericardiocentesis		1	0
Peritonsillar abscess draining		1	0
Thoracentesis		1	0
Nerve blocks		1	0
Suprapubic catheterization		1	0
Deep abscess draining		1	0
Pacemaker installation		1	0
Foreign body removal		1	0

9. How high do you think your skill in these procedures is? Answer every line.

Procedure	Very poor	Poor	Neither poor nor goof	Good	Very good	Do not perform the application in question
Arterial cannulation	1	2	3	4	5	0
Paracentesis	1	2	3	4	5	0
Central vein cannulation	1	2	3	4	5	0
Lumbar punction	1	2	3	4	5	0
Abscess draining	1	2	3	4	5	0
Joint draining	1	2	3	4	5	0
Peripheral vein cannulation	1	2	3	4	5	0
Pericardiocentesis	1	2	3	4	5	0
Peritonsillar abscess draining	1	2	3	4	5	0
Thoracentesis	1	2	3	4	5	0
Nerve blocks	1	2	3	4	5	0
Suprapubic catheterization	1	2	3	4	5	0
Deep abscess draining	1	2	3	4	5	0
Pacemaker installation	1	2	3	4	5	0
Foreign body removal	1	2	3	4	5	0

10. Please evaluate the following statements. Answer every line.

Statements	Strongly disagree	Disagree	Neither agree nor disagree	Agree	Strongly agree
I have an ultrasound machine at my immediate disposal in the emergency department	1	2	3	4	5
I have time to practice ultrasound during most of my shifts	1	2	3	4	5
Performing ultrasound exams is a convenient part of my current job	1	2	3	4	5
I am satisfied with the ultrasound education I have received during my residency	1	2	3	4	5
Ultrasound exams in the emergency department should primarily be performed by emergency physicians instead of radiologists	1	2	3	4	5
I use a hand-held ultrasound machine as conveniently as a stethoscope	1	2	3	4	5
Should I have both a hand-held ultrasound machine and a wheel-on ultrasound machine at my disposal, mainly I choose a hand-held ultrasound machine	1	2	3	4	5

11. Which of the following statements contribute to your use of ultrasound? Answer every line.

Statements	Strongly disagree	Disagree	Neither agree nor disagree	Agree	Strongly agree
My strong theoretical knowledge	1	2	3	4	5
My strong trust on my own findings	1	2	3	4	5
Other physicians consider the quality of my ultrasound exams high	1	2	3	4	5
I get regular feedback from a senior physician	1	2	3	4	5
I have a good possibility to follow exams performed by other physicians	1	2	3	4	5

12. How would you like improve the teaching of ultrasound in your residency?

Thank you for your response!

All information supplied will be treated confidentially.
